# Structure and activity of particulate methane monooxygenase arrays in methanotrophs

**DOI:** 10.1038/s41467-022-32752-9

**Published:** 2022-09-05

**Authors:** Yanan Zhu, Christopher W. Koo, C. Keith Cassidy, Matthew C. Spink, Tao Ni, Laura C. Zanetti-Domingues, Benji Bateman, Marisa L. Martin-Fernandez, Juan Shen, Yuewen Sheng, Yun Song, Zhengyi Yang, Amy C. Rosenzweig, Peijun Zhang

**Affiliations:** 1 Division of Structural Biology, Wellcome Trust Centre for Human Genetics, University of Oxford, Oxford, UK; 2 Departments of Molecular Biosciences and of Chemistry, Northwestern University, Evanston, IL USA; 3 Department of Biochemistry, University of Oxford, Oxford, UK; 4 Diamond Light Source, Harwell Science and Innovation Campus, Didcot, UK; 5 Central Laser Facility, Science and Technology Facility Council, Rutherford Appleton Laboratory, Didcot, Oxfordshire UK; 6 Chinese Academy of Medical Sciences Oxford Institute, University of Oxford, Oxford, UK; 7 Present Address: Imaging Centre, European Molecular Biology Laboratory, Heidelberg, Germany

**Keywords:** Cryoelectron tomography, Air microbiology, Cryoelectron microscopy, Enzymes, Organelles

## Abstract

Methane-oxidizing bacteria play a central role in greenhouse gas mitigation and have potential applications in biomanufacturing. Their primary metabolic enzyme, particulate methane monooxygenase (pMMO), is housed in copper-induced intracytoplasmic membranes (ICMs), of which the function and biogenesis are not known. We show by serial cryo-focused ion beam (cryoFIB) milling/scanning electron microscope (SEM) volume imaging and lamellae-based cellular cryo-electron tomography (cryoET) that these ICMs are derived from the inner cell membrane. The pMMO trimer, resolved by cryoET and subtomogram averaging to 4.8 Å in the ICM, forms higher-order hexagonal arrays in intact cells. Array formation correlates with increased enzymatic activity, highlighting the importance of studying the enzyme in its native environment. These findings also demonstrate the power of cryoET to structurally characterize native membrane enzymes in the cellular context.

## Abstract (web summary)

Particulate methane monooxygenase (pMMO) is the main enzyme used by methanotrophs. Here, the authors determined the native structure of pMMO by cryo-electron tomography, revealing lipid-stabilized features and a higher-order hexagonal array arrangement in intact cells.

## Introduction

Methanotrophs, microbes that utilize methane from the environment as a carbon source^1^, substantially reduce atmospheric methane concentrations and represent a promising platform for biomanufacturing^2,3^. Activation of the 105 kcal/mol methane C-H bond and oxidation to methanol is accomplished by methane monooxygenase (MMO) enzymes at ambient temperature and pressure. Some methanotrophs switch between using the iron-dependent soluble MMO (sMMO)^4^ and the copper-dependent particulate (membrane-bound) MMO (pMMO)^5^ as a function of copper availability^6,7^. MMOs are so critical to the methanotroph lifestyle that they are naturally overexpressed: sMMO constitutes ~10% of the total cellular protein^8^ under copper-starvation conditions, and pMMO represents ~20% of the total cellular protein^9^ and 80% of the total protein in the membranes^10^ under copper-replete conditions.

To accommodate such large quantities of pMMO, copper triggers the formation of intracytoplasmic membranes (ICMs) that fill the cell^11^. These methanotrophic ICMs, detected more than 50 years ago^12^, have been imaged using electron^7,11,13,14^ and fluorescence^15^ microscopies and cryo-electron tomography (cryoET)^16^, but their mechanism of biogenesis is not known. In addition, the arrangement of pMMO within the ICMs and the relationship between this environment and pMMO function remain unclear. Although negative-stain electron microscopy (EM) images of *Methylococcus capsulatus* (Bath) membrane preparations show tight packing of pMMO molecules^17,18^, pMMO has never been directly observed in ICMs within an intact cell.

Structural studies of pMMO have focused on a single biological assembly: a 300 kDa trimer comprising three copies each of subunits PmoA, PmoB, and PmoC. Crystal structures of detergent-solubilized pMMO from five different methanotrophs revealed the overall subunit folds and possible locations for the copper active site, but several transmembrane regions, including a highly conserved part of the PmoC sequence, were not observed^19–23^. This region was finally unveiled in recent single-particle cryo-electron microscopy (cryoEM) structures of pMMO embedded in native lipid nanodiscs, along with a previously-undetected copper site and structural elements stabilized by lipids^18^. The methane oxidation activity of pMMO is also sensitive to solubilization from the ICMs. From whole cells to isolated membranes, catalytic activity decreases by 5–10 fold^24^, and upon solubilization in detergent, there is a further 10-fold reduction in activity^25^ with no measurable activity for crystallized samples^19^. Some activity is recovered in lipid nanodiscs, allowing the cryoEM structure determination of enzymatically-active pMMO, but this recovered activity is less than that of isolated membranes and far below whole cell levels^18,23^. The inability to fully recover methane oxidation activity is potentially attributable to pMMO removal from the unique ICM environment.

In contrast to X-ray crystallography and single-particle cryoEM, cryoET offers the opportunity to study pMMO in its most active state in the cell and in isolated ICMs, without perturbation by detergent solubilization or non-native environments. CryoET with subtomogram averaging (STA), combined with cryo-focused ion beam (cryoFIB) milling, provides a powerful approach for studying molecular complexes in situ^26,27^, and has been applied successfully to a variety of intact bacterial cells and reconstituted bacterial systems^28–31^. In addition, the entire volume of a vitrified cell can be reconstructed at tens of nanometers resolution through serial cryoFIB/SEM imaging^32,33^. Here we have used these techniques to investigate pMMO assembly and structure as well as ICM biogenesis in intact *M. capsulatus* (Bath) methanotroph cells and isolated native membranes. The data indicate that the ICMs derive directly from the inner cell membrane, where pMMO assembles into an ordered hexagonal array. The cryoET STA structure of the pMMO trimer embedded in the ICM was determined to 4.8 Å resolution, and is consistent with the structure in native nanodiscs^18^. The observed hexagonal arrays of pMMO trimers provides insights into the high enzymatic activity of pMMO in native cells.

## Results

### Biogenesis of intracytoplasmic membranes and pMMO arrays in intact cells

**Fig. 1:**
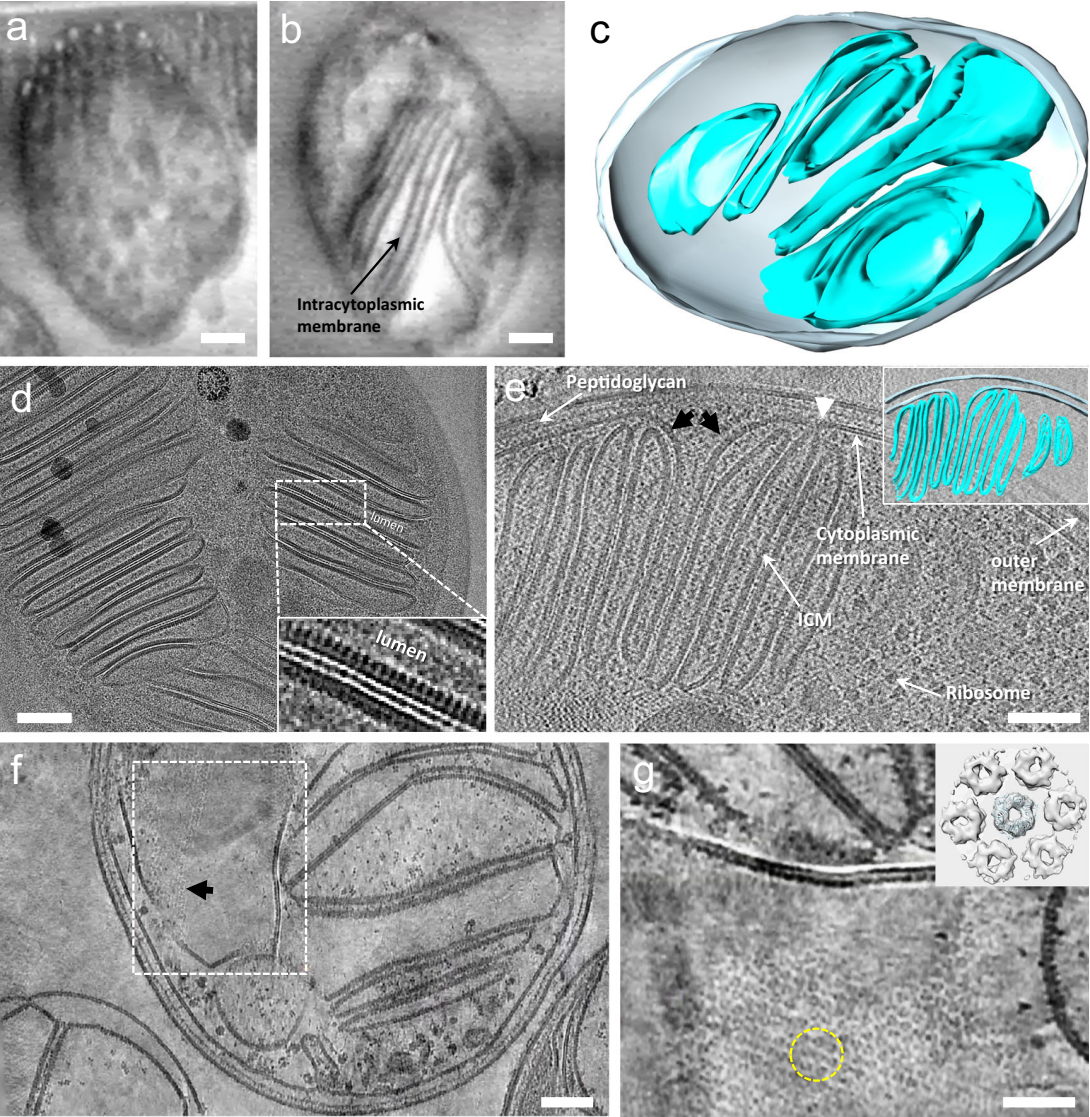
Copper-induced ICMs of methanotrophic bacteria. **a, b** Representative slices from serial cryoFIB/SEM volumes of *M. capsulatus* (Bath) cultured in a medium containing 0 μM (**a**) or 25 μM (b) CuSO_4_. For (**a**), similar volume images were conducted in five independent experiments. For (**b**), similar volume images were conducted in ten independent experiments. **c** Membrane segmentation of the cell in (**b**). Cell membrane is shown in grey, and ICMs are shown in cyan. **d** A projection image of cryoFIB lamella of *M. capsulatus* (Bath) cultured in the copper-containing medium (from *n* = 20). Inset, enlarged view of boxed area, displaying comb-like edge-on view of membrane-bound protein arrays. **e** An 8.5 nm thick tomographic slice of *M. capsulatus* (Bath) cell lamella (from *n* = 20). Inner and outer cell membranes, peptidoglycan, ICM, and ribosome are labeled. The inner cell membrane continues with ICM (black arrows), where the transition point is marked (white arrowhead). **f** A tomographic slice of *M. capsulatus* (Bath) lamella displaying an array of particles (black arrow) (from *n* = 10). **g** An enlarged view of the boxed region in (**f**), tilted and rotated to show the face-on view of membrane-bound particles organized in a hexagonal lattice (from *n* = 10). Inset, a subtomogram average of the 7-particle volume (yellow circle). Scale bars, 200 nm in (**a**, **b**), 100 nm in (**d**) to (**f**), and 50 nm in (**g**).

To investigate ICM biogenesis, we vitrified *M*. *capsulatus* (Bath) cells grown in the presence and absence of copper and imaged them by serial cryoFIB/SEM for whole bacterial cell volumes. The reconstructed 3D volumes of intact cells show abundant ICM stacks in cells cultured in copper-containing media, but not in cells cultured without copper (Fig. 1a–c, Supplementary Movie 1–2), consistent with previous observations^7,11,13–15^. We then used cryoFIB to create thin cell lamellae (<200 nm) from plunge-frozen cells cultured in the presence of copper (Fig. 1d–g). Projection images of thin cell lamellae show extensive ICM stacks filled with pMMO particles, which appear like fine tooth combs when viewed along the membrane surface with the soluble domain facing the lumen (Fig. 1d and inset). In the reconstructed cryo-tomogram of the cell lamella, the outer membrane, peptidoglycan cell wall, and cytoplasmic membrane are distinct (Fig. 1e–f). The ICM vesicles appear separated from each other (Fig. 1e inset, Supplementary Movie 3). There is a clear continuity from the cytoplasmic membrane to the ICM (Fig. 1e black arrows), indicating that the ICMs are likely derived from the cytoplasmic membrane. Moreover, a transition point where the membrane (with a rough periplasmic side) differentiates from the cytoplasmic membrane (displaying two leaflets of the bilayer) can be identified (Fig. 1e white arrowhead). Although a static image or 3D volume is not sufficient to distinguish between invagination of ICM and a fusion of ICM disk with inner membrane, it is more likely that the ICMs containing fully embedded pMMO trimers are invaginated from inner membrane when pMMO starts to insert into membrane than fusion of ICM disk with inner membrane in which case one would expect to observe pMMO arrays also in the inner membrane. The fluorescence microscopy data^15^ also support this. These observations clearly demonstrate that ICMs form by invagination of the cytoplasmic membrane under copper-replete conditions^15,34,35^.

Most lamellae display the cross-section of ICM vesicles where pMMO particles were seen densely packed like fine tooth combs (Fig. 1d, e). However, lamellae that were milled along the ICMs surprisingly exhibit highly-ordered hexagonal arrays of pMMO particles covering the whole membrane surface (Fig. 1f–g, Supplementary Movie 4). Such extensive ordered arrays within intact methanotrophs have never been observed. Extracting and aligning subtomogram volumes containing seven particles (Fig. 1g, dashed yellow circle) yielded a subtomogram averaged map at 15 Å resolution (Fig. 1g inset). The map reveals donut-shaped particles with a central particle surrounded by six equivalent neighbours. The central particle can be fitted well with the pMMO trimer crystal structure (PDB 3RGB)^21^ (Fig. 1g inset).

### CryoET STA structure of native pMMO trimer in ICMs

**Fig. 2:**
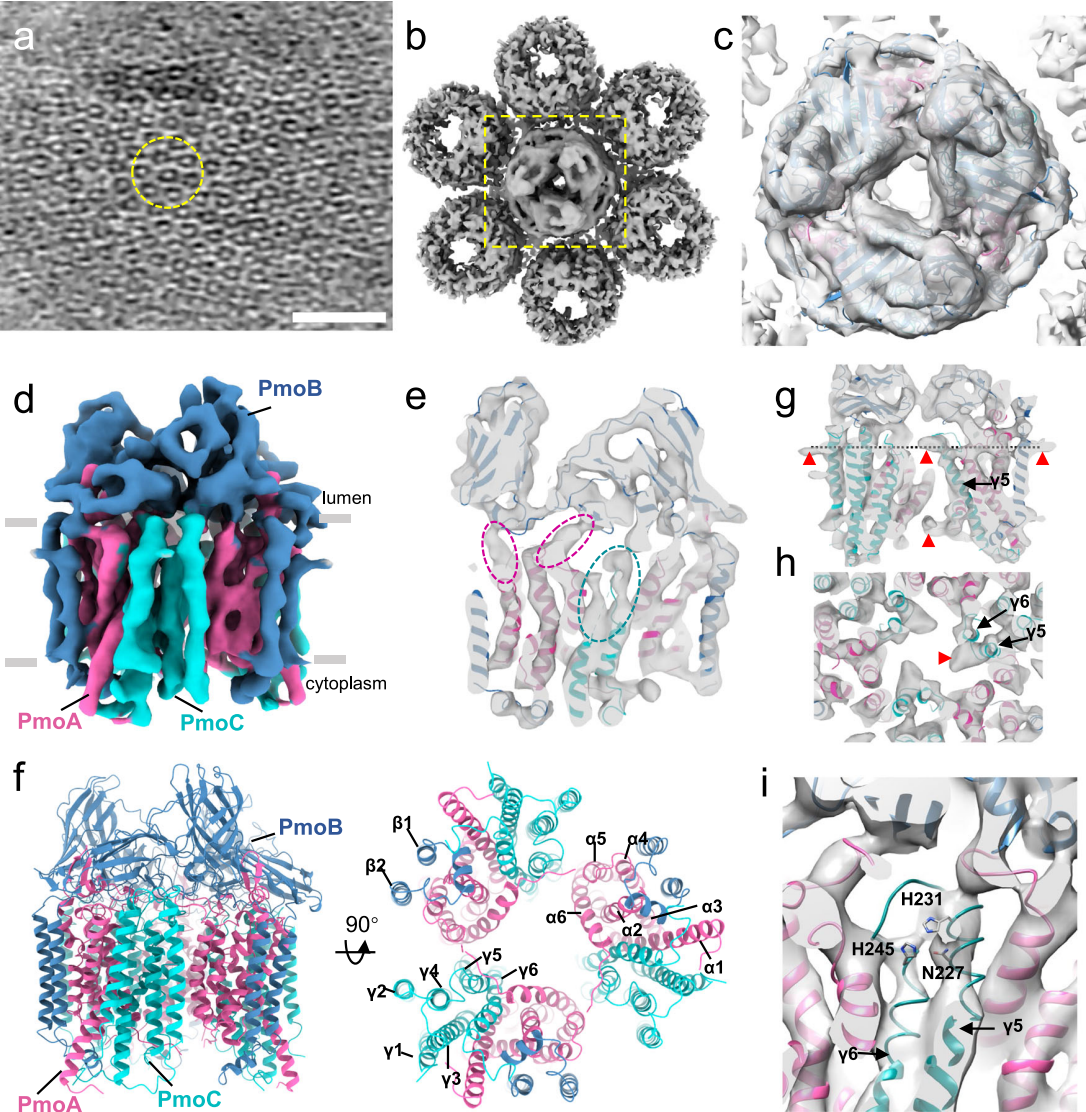
CryoET STA of native pMMO trimer from isolated ICMs. **a** A tomographic slice of isolated ICM (from *n* = 187). A yellow dashed circle encloses a unit of 7 particles. **b** Subtomogram average of the 7-particle unit. **c** An enlarged view of the central particle in (**b**), superimposed with the docked crystal structure of the pMMO trimer (PDB 3RGB). **d** The pMMO trimer STA map at 4.8 Å resolution, with each pMMO monomer consisting of PmoA (pink), PmoB (blue), and PmoC (cyan). **e** A central slice view of pMMO map superimposed with pMMO crystal structure model. The dashed oval regions indicate the densities for PmoA (pink) and PmoC (cyan) that were not resolved in the previous crystal or cryoEM SPA structures of detergent-solubilized pMMO. **f** Overall structure of active pMMO trimer embedded in a native membrane, with PmoA, PmoB, and PmoC colored accordingly. Alpha helices of PmoA, PmoB, and PmoC are labeled as α1-6, β1-2, and γ1-6, respectively. **g, h** Side and cross-sectional (at the plane indicated by the dotted line) views of the pMMO map, superimposed with the cryoEM pMMO nanodisc model (PDB 7S4H). Red arrowheads indicate extra densities that are not fitted with the model. **i** Three invariant residues, N227, H231, and H245 from PmoC helices γ5 and γ6, bind copper in the cryoEM pMMO nanodisc model (PDB 7S4H). Scale bars, 50 nm.

To understand the molecular organization of the extended array, we isolated membranes from *M. capsulatus* (Bath) and carried out cryoET analysis. CryoEM projection images show that the membranes are densely packed with small ring-like particles similar to those observed in the intact cell (Supplementary Fig. 1b), which are presumably pMMO trimers as the pMMO subunits are the main components in the sample (Supplementary Fig. 1a) and the particle size and shape are consistent with the pMMO crystal and cryoEM structures. CryoET of these membrane vesicles reveals that the ring-like particles are organized into an ordered hexagonal array (Fig. 2a, Supplementary Movie 5). The distance between two neighboring particles is ~10 nm, similar to the interparticle distance in the cell (Fig. 1g). Using a template containing seven pMMO particles (Fig. 2a, dashed yellow circle), the result of template matching by emClarity^36,37^ reveals that more than 70% of membranes are covered by the hexagonal array of pMMO particles (Supplementary Fig. 1c). Further subtomogram averaging resulted in a map at an overall resolution of 10 Å, where the central particle is well resolved (Fig. 2b) and fits the crystal structure of pMMO trimer neatly (Fig. 2c). In contrast to the relatively equal quality among the seven particles derived from cell lamella (Fig. 1g inset), the central particle here is much better resolved than the surrounding six particles, indicating that the hexagonal lattice in these isolated ICMs is less well ordered compared to that in the intact cell. Disruption of the lattice may contribute to the reduced catalytic activity upon membrane isolation^24^.

Previous structural studies of pMMO involved detergent solubilization steps that abolish or diminish activity, including purification in detergent for crystallization^19–23^ and reconstitution of detergent-solublized samples into lipid nanodiscs for cryoEM single particle analysis^18^. To investigate pMMO in its native environment without the use of detergent, we conducted cryoET STA of pMMO embedded within isolated *M. capsulatus* (Bath) membranes. We determined the structure of the pMMO trimer to 4.8 Å resolution (Fig. 2d, Supplementary Fig. 2, Supplementary Table 1), unprecedented for integral membrane protein complexes by cryoET^38–42^ (Supplementary Table 2). The trimer architecture and folds of PmoA, PmoB, and PmoC resemble the previous crystal and cryoEM structures. Densities corresponding to regions of PmoA and PmoC not resolved crystallographically are apparent in the map, including part of PmoA (Fig. 2e, dashed pink ovals) and segments of two highly conserved PmoC helices facing the interior of the trimer (M219 to L254) (Fig. 2e, dashed cyan oval). These regions were fully modelled in the recent cryoEM structure of pMMO in native nanodiscs^18^, which fits the cryoET STA map well (Fig. 2f, Supplementary Fig. 2, Supplementary Movie 6). Several densities not accounted for by the pMMO model are also present and potentially correspond to lipids (Fig. 2g, h). The high resolution of the native nanodisc cryoEM structure (2.14 Å) (PDB 7S4H) also allowed the modelling of a copper site coordinated by PmoC residues Asn 227, His 231, and His 245^18^. Although the side chains are not resolved at 4.8 Å resolution, density corresponding to the backbone of the helices is present in our cryoET STA map from native ICMs (Fig. 2i).

### pMMO higher-order array supports high pMMO activity

**Fig. 3:**
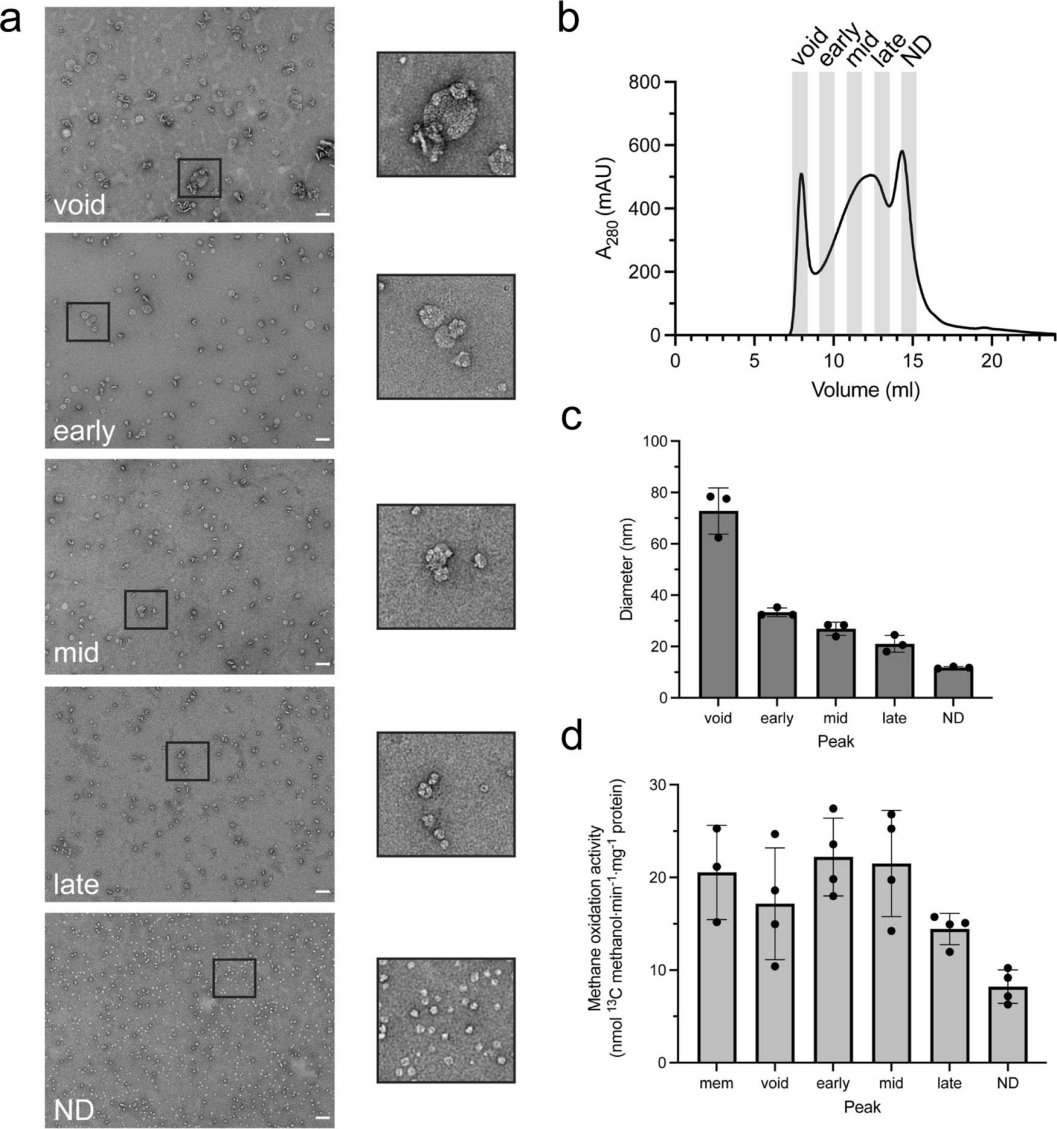
Methane oxidation by pMMO reconstituted in native lipid nanodiscs. **a** Representative images and enlarged views of the boxed area from elution fractions of pMMO nanodiscs separated on a Superose 6 size-exclusion column. Nanodiscs were generated using a ratio of 1:2:120 pMMO:MSP1E3D1:lipid. Similar micrographs were obtained in three independent experiments. **b** The elution profile of pMMO nanodisc size-exclusion column. The fractions that were collected are indicated. **c** Size distribution of pMMO nanodiscs measured by dynamic light scattering. *n* = 3 biologically independent samples. Data are presented as mean values + /− the standard deviation. **d** pMMO specific activities were measured from each fraction and isolated membranes. *n* = 4 biologically independent samples. *n* = 3 biologically independent samples were used for membrane (mem) activity. Data are presented as mean values + /− the standard deviation. Scale bar, 50 nm. Source data are provided as a Source Data file.

CryoET of both cryoFIB milled intact cells and isolated ICMs reveals that pMMO assembles into regular hexagonal arrays in the native membrane (Fig. 1g, Fig. 2a). Furthermore, when purified pMMO was reconstituted into lipid nanodiscs, we observed a substantial fraction of nanodiscs containing three pMMO trimers in samples that were optimized for formation of single pMMO trimer-containing nanodiscs (Supplementary Fig. 3). These findings raise a key question of whether the pMMO higher-order array plays a role in driving ICM biogenesis and high pMMO activity in native cells^24^. To investigate the relationship between array formation and methane oxidation, we formed arrays with native lipid nanodiscs and measured the resulting activity. Previous work with nanodiscs has shown that altering the ratio of lipid to membrane scaffold protein (MSP) can result in larger nanodiscs containing more than one enzyme per disc^43,44^. When we reconstituted pMMO into nanodiscs with a 1.5 x lipid:MSP ratio compared to the ratio optimized for single particle nanodiscs, we were able to separate nanodiscs of various sizes using size-exclusion chromatography (SEC) (Fig. 3a, b). The SEC fractions exhibited decreasing size with elution volume, with the single particle nanodiscs measuring ~100 Å by dynamic light scattering (Fig. 3c) as expected based on the cryoEM single particle structure^18^. Small partially ordered patches of pMMO assemblies were observed by negative stain EM (Fig. 3a). Interestingly, the SEC fractions corresponding to the larger nanodisc arrays exhibited up to 3-fold the methane oxidation activity of the single particle nanodiscs, reaching activity levels close to that of pMMO in isolated membranes (Fig. 3d). Although the array formation in nanodiscs may not be exactly the same as that in the ICM, these observations support the idea that pMMO activity is enhanced by array formation.

### PmoB mediates pMMO hexagonal array assembly

**Fig. 4:**
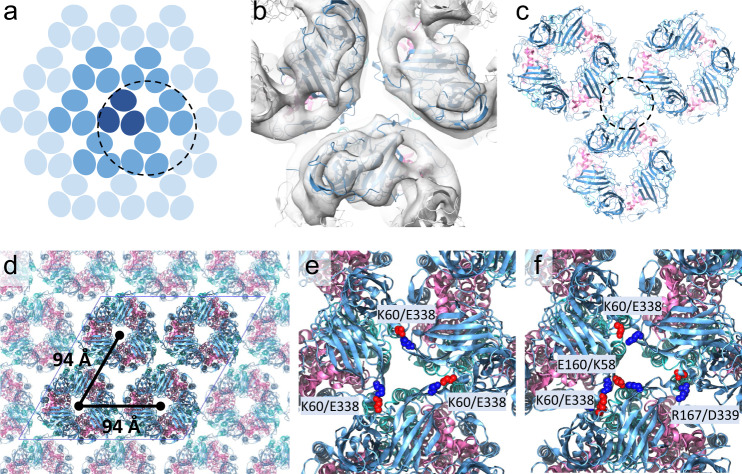
Higher-order assembly of pMMO trimers. **a** A schematic of the pMMO trimer hexagonal array. Each blue oval represents a pMMO monomer consisting of PmoA, PmoB, and PmoC. The central pMMO trimer (dark blue), together with 6 surrounding trimers (medium blue), represent the 7-particle unit shown in Fig. 1g and Fig. 2b. **b** CryoET STA of the subvolumes enclosing an inter-trimer interface, shown as a circled unit in (**a**), superimposed with docked pMMO model. (**c**) Structural model of pMMO trimer assembly. The inter-trimer interaction is mediated by PmoB (blue), shown in the dashed circle. **d** Molecular model of the extended pMMO trimer hexagonal array, with a 94 Å inter-trimer distance. The four-trimer system used for MD simulation is shown in opaque colors with the simulation box bounded by a blue rhombus and periodic pMMO trimer images shown as transparent. PmoA, PmoB, and PmoC colors are as in Fig. 3. **e**, **f** Interactions between the neighboring pMMO trimers, mediated by pairs of charged residues, K60/E338, K58/E160, and R167/D339, as indicated.

To further investigate array formation, we focused our cryoET STA analysis on the inter-trimer interface (Fig. 4a), obtaining a 12 Å resolution STA map into which three pMMO trimers were fit unambiguously using rigid-body docking (Fig. 4b, c). We then used all-atom molecular dynamics simulation to identify specific residues mediating trimer-trimer interactions. For this purpose, we constructed a 2 × 2 lattice patch containing four pMMO trimers (Fig. 4d), utilizing periodic boundary conditions to mimic the symmetry of the extended pMMO array. Analysis of the four distinct trimer interfaces contained within the system over a 100-ns simulation showed several interactions between the PmoB soluble domains of adjacent pMMO trimers, including a strong salt bridge interaction between K60 and E338 (Fig. 4e, Supplementary Fig. 4) as well as more transient interactions involving residues K58/E160 and R167/D339 (Fig. 4f, Supplementary Fig. 4). These residues comprise interfacial clusters with complementary charges. There is a moderate degree of conservation for K60 and E338, while other pairs are less conserved (Supplementary Fig. 5). No direct protein interactions were observed within the transmembrane domains of PmoA, PmoB, or PmoC, suggesting that the inter-trimer contacts responsible for array formation are primarily mediated by the PmoB soluble domains.

## Discussion

Recent work has shown that studying pMMO in as close to the native lipid environment as possible is required for a thorough understanding of its function^18,23,45^. Our cryoET STA investigation of pMMO in intact cells and isolated membranes reveals that pMMO forms hexagonally ordered arrays in ICMs that derive directly from the inner cell membrane. The formation of these arrays, which are possibly stabilized by salt-bridge interactions between PmoB subunits, correlates with increased methane oxidation activity, explaining in part why pMMO activity decreases substantially upon disruption of the membranes by detergent solubilization and cannot be fully recovered in single particle native lipid nanodiscs^18^. In vitro, the arrays might enhance activity by increasing the solubility of methane, oxygen, and the duroquinol reductant used in the assay. In the cell, the formation of dense pMMO arrays in the inner membrane might trigger ICM formation and serve to exclude other enzymes from the respiratory pathway from the ICMs. The arrays might also facilitate recruitment of periplasmic methanol dehydrogenase (MDH) to more efficiently link methane oxidation by pMMO to the next step in the metabolic pathway, methanol oxidation to formaldehyde by MDH^46,47^. Understanding how pMMO arrays assemble and promote methane oxidation will be integral to future efforts to deploy methanotrophs in biotechnology.

## Methods

### Cell culture and membrane isolation

Copper-replete *Methylococcus capsulatus* (Bath) was cultured in a 12 L reactor with a continuous flow of 1:3 methane:air at 1 L/min and 45 °C. The culture was grown as described previously in nitrate mineral salts (NMS) buffer containing KNO_3_, MgSO_4_·7H_2_O, CaCl_2_·2H_2_O. 3.9 mM phosphate buffer, pH 6.8, trace element solution, 80 μM NaFeEDTA, and 50 μM CuSO_4_·5H_2_O^19^. Cells were grown to an optical density at 600 nm (OD_600_) of 7.0, harvested by centrifugation at 5000 x g for 10 min, flash frozen, and stored at −80 °C.

Copper-starved *M. capsulatus* (Bath) was first grown in 50 ml cultures for 2 weeks with 10 μM CuSO_4_ and then passaged into another 50 ml culture for 2 weeks at 0.5 μM CuSO_4_. This culture was then used to inoculate a 3 L bioreactor culture with 0 μM CuSO_4_ for 1 week until OD_600_ of 5.0 was reached. Cells were harvested as described above, flash frozen, and stored at −80 °C.

To isolate membranes, cells were thawed in buffer containing 25 mM PIPES, pH 7.3, 250 mM NaCl with DNase and protease inhibitor cocktail (Roche). The cells were sonicated on ice for 10 min at 40 % amplitude with 1 s /1 s on/off pulses (Qsonica 700). The lysate was centrifuged at 20,000 xg for 45 min followed by collection of the soluble fraction and centrifugation at 140,000 xg for 1 h at 4 °C to isolate the membranes. The isolated membranes were washed with 40 ml of the same buffer, passing through a Dounce homogenizer before centrifuging again. This step was repeated for a total of three washes. After the final spin, membranes were resuspended in 8 ml of the same buffer, aliquoted, and flash frozen. The protein concentration in the membranes was measured by the DC Lowry assay (Bio-Rad).

### pMMO solubilization, nanodisc reconstitution, and activity measurements

pMMO was isolated from membranes using n-dodecyl-β-D-maltoside (DDM) mixed with the membranes at a 1.2:1 mg:mg ratio at 4 °C for 1 h. The mixture was then centrifuged at 140,000 x g to separate out the insoluble portion. Solubilized pMMO was concentrated and exchanged into buffer containing 25 mM PIPES, pH 7.3, 250 mM NaCl, 0.02% DDM. The protein concentration was measured using the DC-Lowry assay (Bio-Rad).

To form nanodiscs of various sizes, pMMO was mixed with lipids isolated from the native organism and membrane scaffold protein (MSP1E3D1), both prepared as described previously^18^. A molar ratio of 1:2:120 pMMO:MSP1:lipid was used to generate these nanodiscs; a larger amount of lipid than necessary was used to ensure formation of homogeneous nanodiscs. The mixture was rotated end-over-end at 4 °C for 15 min along with 20 mM sodium cholate before adding 0.8 mg/ml SM-2 Bio-Beads (Bio-Rad). After 2 h, the Bio-Beads were removed using a syringe filter, and the formed discs were purified using a Superose 6 column (Cytiva). Fractions were collected from the void, early, mid, late, and nanodisc (ND) peaks for further characterization (Fig. 3). MSP2N2 discs were generated by the same method using a ratio of 1:1:360 pMMO:MSP2:lipid.

Dynamic light scattering analysis of fractions was performed with a Punk instrument (Unchained Labs), analyzing 5 μl of sample over 10 s with five repeats per sample. Negative-stain samples were prepared by applying 3 μl sample onto a 400-mesh copper grid. Grids were washed for two rounds of 15 s with water and two rounds of 30 s with 1% uranyl formate. Grids were then blotted and allowed to dry. Images were collected on a JEM-1400 microscope (JEOL) at 30000x magnification at 3.71 Å pixel size using Leginon software.

Activity assays were carried out by adding 100 μl sample containing 2 mg/ml pMMO to a screw-top vial, adding an excess amount of duroquinol, removing 1 ml headspace, adding 1.5 ml ^13^C-methane and shaking at 45 °C for 5 min. Negative control reactions were performed omitting enzyme, duroquinol, and methane. Reactions were stopped by storing at −20 °C. To analyze the methanol produced by the reactions, the samples were thawed and 500 μl chloroform with 1 mM dicholoromethane was added to each sample as an internal standard. Samples were shaken at 2000 rpm at 4 °C for 10 min followed by centrifugation at 2000 x g for 10 min at 4 °C. Samples were then analyzed for ^13^C-methanol concentration using an Agilent 7890B/5977 A MSD GC/MS instrument with a PoraBOND Q column (25 m x 250 μm x 3 μm) according to previous methods^23,45^. The ^13^C-methanol peak was integrated, compared to a standard curve, and normalized to the concentration of dichloromethane.

### CryoET sample preparation

For *M*. *capsulatus* (Bath) bacteria, the cells were diluted to an OD_600_ of 2 with NMS buffer. Quantifoil grids (300 mesh, Cu R2/1) were glow-discharged for 45 s. 4 μl of the sample were added onto the carbon side of the grid, which were blotted from the mesh side for 10 s before plunge freezing into liquid ethane with a Leica GP2 (Leica Microsystems). The humidity was set to 100% and the temperature was set to 15 °C during blotting.

For the isolated *M*. *capsulatus* (Bath) membranes, a 5 mg/ml stock membrane solution was diluted to 1.25 mg/ml with buffer containing 25 mM PIPES, pH 7.3 and 250 mM NaCl, and mixed with 6 nm gold beads before plunge-freezing. Lacey carbon-coated copper grid (300 mesh, Agar Scientific) was glow-discharged for 45 s. 3 μl of the sample were added onto the carbon side of the grid, and the grid was blotted for 3.5 s with a blotting force of −15 before plunge freezing into liquid ethane using a Vitrobot (Thermo Fisher Scientific). The humidity was set to 100% and the temperature was set to 4 °C during blotting.

### Serial CryoFIB/SEM

Samples were imaged on a ZeissCrossbeam 550XL (Carl Zeiss Microscopy, LLC), fitted with a Quorum transfer station and cryo-stage (Quorum Technologies). They were mounted on a Quorum-compatible custom sample holder and coated with Pt for 60 s at 10 mA on the Quorum transfer stage, before loading on the cryo-stage. The stage temperature was set at −165 °C, while the anticontaminator was held at −185 °C. Samples were imaged at 45° tilt after being coated again with Pt for 2x 30 s using the FIB-SEM’s internal GIS system, with the Pt reservoir held at 25 °C.

Initial trapezoid trenches were milled at 30 kV 700 pA over 8.5 μm to reach a final depth of 3.5 μm, without a further polishing step. Serial sectioning and imaging acquisition was performed as follows: for high Cu samples, FIB milling was performed using the 30 kV 50 pA probe, while for low Cu samples, it was done at 30 kV 20pA, and both were acquired with a z-slice step of 10 nm and a depth of 3.5 μm over the entire milling box of 8 × 8 μm; SEM imaging was performed at a pixel depth of 2048 ×1 536 pixels, which resulted in a pixel size of 5 nm, with the beam set at 1.5 kV 20pA, dwell time 50 nsec and scan speed 0, averaging the signal over 100 line scans as a noise-reduction strategy.

### CryoFIB milling for lamella preparation

Lamella preparation of *M*. *capsulatus* (Bath) was performed using a Scios DualBeam cryoFIB/SEM (Thermo Fisher Scientific) equipped with a PP3010T transfer system and stage (Quorum Technologies) using the xT v7.6 software (Thermo Fisher Scientific). Grids were sputter coated with Pt within the PP3010T transfer chamber maintained at −175 °C. After loading onto the Scios stage at −168 °C, the grids were inspected using the SEM (operated at 5 kV and 13 pA). The grid surface was coated using the gas injection system trimethyl (methylcyclopentadienyl) platinum (IV) (Thermo Fisher Scientific) for 4 s, to give a thickness of ~3 µm. Milling was performed using the ion beam operated at 30 kV and currents decreasing from 300 to 30  pA. At 30 pA, lamella thickness was thinned to < 200 nm. During the final stage of milling, SEM lamella inspection was conducted at 2 kV and 13 pA.

### CryoET data collection

For isolated membranes, cryoET tilt series were acquired using a Thermo Fisher Titan Krios operated at 300 keV equipped with a Gatan Quantum post-column energy filter (Gatan Inc) operated in zero-loss mode with 20 eV slit width, and Gatan K3 direct electron detector in eBIC (electron BioImaging Centre, Diamond). Tilt series were collected with SerialEM^48^ with a nominal magnification of 64k and a physical pixel size of 1.34 Å per pixel. They were acquired using dose-symmetric tilt-scheme^49^ starting from 0° with a 3° tilt increment by a group of three and an angular range of ± 63°. The accumulated dose of each tilt series was around 129 e^-^/Å^2^ with a defocus range between −2.5 and −6 µm. Ten frames were saved in each raw tilt image. Details of data collection parameters are listed in Supplementary Table 1.

For cell lamellae, cryoET tilt series were collected with SerialEM^48^ with a nominal magnification of 42k and physical pixel size of 2.13 Å/pix. They were acquired usng dose-symmetric tilt-scheme^49^ starting from 0° with a 3° tilt increment by a group of three and an angular range of ± 54°. The accumulated dose of each tilt series was around 111 e^-^/Å^2^ with a defocus range between −3.5 and −6 µm. Ten frames were saved in each raw tilt image.

### CryoET data processing and subtomogram averaging

The automated cryoET pipeline developed in-house was used for initial tomograms (https://github.com/ffyr2w/cet_toolbox) through performing motion correction^50^ of the raw frames, tilt-series alignment and final reconstruction with IMOD^51^. Suitable tilt series were selected. For isolated membranes, the fiducial markers were manually inspected to ensure the centering of predicted markers for each tilt series in eTOMO. For cell lamellae, a fiducial-less tilt-series alignment was performed by using AreTomo^52^.

Subtomogram averaging from the isolated intracytoplasmic membranes was performed following the workflow of emClarity^36,37^. The pMMO trimer crystal structure (PDB 3RGB)^21^ was low-pass filtered to 30 Å and used as the initial template for template search. A small dataset of 10 tilt series was used for template search with 4x binned tomograms with a pixel size of 5.36 Å. About 2,000 subtomograms were selected based on their initial positions and orientations in the membrane. Subtomogram averaging and alignment were performed with a volume including 7-pMMO particles. After several refinement cycles, the density map with 7-pMMO was obtained.

A further 127,417 subtomograms were selected from the entire dataset of 187 tilt series. The subtomogram averaging and alignment were performed iteratively using 3x and 2x binned tomograms. A cylindrical alignment mask including one pMMO particle and a 3-fold symmetry were used throughout the alignment procedure. We converted the coordinates from emClarity to Relion-4.0^53^ for Relion 3D refinement in bin3 (4.02 Å pixel size) and bin2 (2.68 Å pixel size). The final density map was reconstructed at bin 1 with Relion-4.0^53^ and sharpened with a b-factor of −50.

For the three-trimer subtomogram averaging, the orientations for each particle from the above step was kept and the position shifted to the centre of three trimers. A cylindrical alignment mask including the central 3 trimers and a 3-fold symmetry were used for additional averaging and alignment in bin 2 (2.68 Å pixel size) in Relion-4.0^53^.

Subtomogram averaging of pMMO from lamellae was processed similarly as the isolated intracytoplasmic membranes using emClarity^36,37^. A template search using the 7-particle reference was carried out in 4x binned tomograms with a pixel size of 8.52 Å for the thin lamellae. A total of 500 subtomograms were selected based on their initial positions and orientations in the intracellular membranes. The subtomogram averaging and alignment were performed iteratively using 3x and 2x binned tomograms, with pixel sizes of 6.39 Å and 4.26 Å. respectively. A cylindrical alignment mask and a 3-fold symmetry were applied throughout the alignment procedure. The final density map was reconstructed at bin 2 with emClarity.

### Model building and refinement and analysis

A starting model of the pMMO trimer was constructed by rigid-body docking the cryoEM SPA structures of PmoA, PmoB and PmoC from pMMO nanodiscs (PDB 7S4H)^18^ into our cryoET STA map using Chimera^54^. A model of the extended pMMO array was constructed by hexagonally tiling the pMMO trimer using a lattice constant of 9.4 nm as determined from the subtomogram average of three pMMO trimers (Fig. 4b, c). A four-trimer region was then manually embedded in a lipid bilayer constructed and equilibrated using CHARMM-GUI^55^ and containing 57% PVPE, 18% PVPG, 16% POPC, and 9% cardiolipin^56^ and subsequently solvated with TIP3P water and 150 mM NaCl using VMD v1.94^57^. The resulting system contained a total of ~420,000 atoms, including ~52,000 water molecules and ~730 lipid molecules, and was placed in a simulation box with dimensions x = y = 187 Å, z = 123 Å and axial angles α = β = 90°, ɣ = 60°.

### Molecular dynamics simulations

All-atom molecular dynamics simulations were carried out using NAMD v2.14^58^ and the CHARMM36 force field^59^.The four-trimer hexagonal patch was first subjected to a series of conjugant gradient energy minimizations followed by a 10-ns equilibration simulation with backbone restraints and finally a 100-ns production simulation without restraints. Simulations were conducted in the NPT ensemble with conditions maintained at 1 atm and 310 K using the Nosé–Hoover Langevin piston and Langevin thermostat respectively. The r-RESPA integrator scheme was employed with an integration time step of 2 fs and SHAKE constraints applied to all hydrogen atoms. Short-range, nonbonded interactions were calculated every 2 fs with a cut-off of 12 Å; long-range electrostatics were evaluated every 6 fs using the particle-mesh-Ewald method. Periodic boundary conditions were appropriately chosen to permit the protein to interact with its periodic image in the x-y plane, mimicking the molecular interactions within the extended hexagonal array.

### Reporting summary

Further information on research design is available in the Nature Research Reporting Summary linked to this article.

## Supplementary information

Supplementary Information

Description of Additional Supplementary Files

Supplementary Movie 1

Supplementary Movie 2

Supplementary Movie 3

Supplementary Movie 4

Supplementary Movie 5

Supplementary Movie 6

Reporting Summary

## Data Availability

The data that support this study are available from the corresponding authors upon reasonable request. The cryoET STA maps have been deposited in the Electron Microscopy Data Bank (EMDB) under accession codes EMD-14399 (single pMMO trimer) and EMD-14530 (three pMMO trimer). Source data underlying Fig. 3c,d and Supplementray Fig. 1a can be found in the Source Data file. Source data are provided with this paper.

## Source data

Source Data
