# Supplementary material for: Structure and activity of particulate methane monooxygenase arrays in methanotrophs
Source: Nat Commun. 2022 Sep 5;13:5221. doi: 10.1038/s41467-022-32752-9 (PMC9445010; doi:10.1038/s41467-022-32752-9)
Supplement: Supplementary file 1 — Supplementary Information [file 41467_2022_32752_MOESM1_ESM.pdf]

Supplementary Information

For

Structure and Activity of Particulate Methane Monooxygenase Arrays in Native Methanotrophs

Yanan Zhu, Christopher W. Koo, C. Keith Cassidy, Matthew C. Spink, Tao Ni, Laura C. Zanetti-Domingues, Benji Bateman, Marisa L. Martin-Fernandez, Juan Shen, Yuewen Sheng, Yun Song, Zhengyi Yang, Amy C. Rosenzweig and Peijun Zhang

List of Supplementary materials

Supplementary Table 1 – 2
Supplementary Figure 1 – 5
Supplementary Movie 1-6

Supplementary Table 1 | CryoET data collection and structure determination of pMMO trimer from isolated intracytoplasmic membranes.

**Data acquisition**	
Microscope	Titan Krios
Voltage (keV)	300
Detector	Gatan Quantum K3 Direct Electron Detector
Energy-filter	Yes
Slit width (eV)	20
Super-resolution mode	No
Å/pixel	1.34
Defocus range (µm)	-2.5 to -6
Defocus increment (µm)	0.3
Acquisition scheme	Dose-Symmetric, -63/63, 3° step, group 3
Total Dose (electrons/Å^2)	129
Number of Frames	10
Number of Tomograms	187
**Structure determination**	
No. of subotomograms	127,417
Resolution (Å)	4.8
b-factor applied	-50
Data deposited	EMD-14399, EMD-14530

Supplementary Table 2 | Summary of EMDB depositions of *in situ* membrane protein structures determined by cryoET STA.

EMDB ID	Res*	Sample information	Ref
EMD-4315	9.1	OST-containing ribosome-translocon complexes from canine rough microsomal membranes	(37)
EMD-8330	6.4	Outer membrane pore complex PilQ in Pelagibacter cell	**
EMD-10840	9.1	Structure of RyR1 in apo/closed state in native membrane	(38)
EMD-10752	7.7	Hexagon bounding clathrin leg in clathrin coats assembled on a membrane	(39)
EMD-10753	7.7	Pentagon bounding clathrin legs in clathrin coats assembled on a membrane	(39)
EMD-21411	9.4	HIV envelope glycoprotein bound with soluble CD4 (D1-D2) and antibody 17b on AT-2 treated BaL strain virus	(40)
EMD-21413	9.9	HIV envelope glycoprotein bound with antibodies 10-1074 and 3BNC117 on AT-2 treated BaL strain virus	(40)
EMD-25178	9.1	STA structure of HIV-1 Envelope protein in native membrane	(41)
EMD-14399	4.8	STA of pMMO trimer in intracytoplasmic membranes	***

* The achieved resolution in Å.
** not published
*** This study

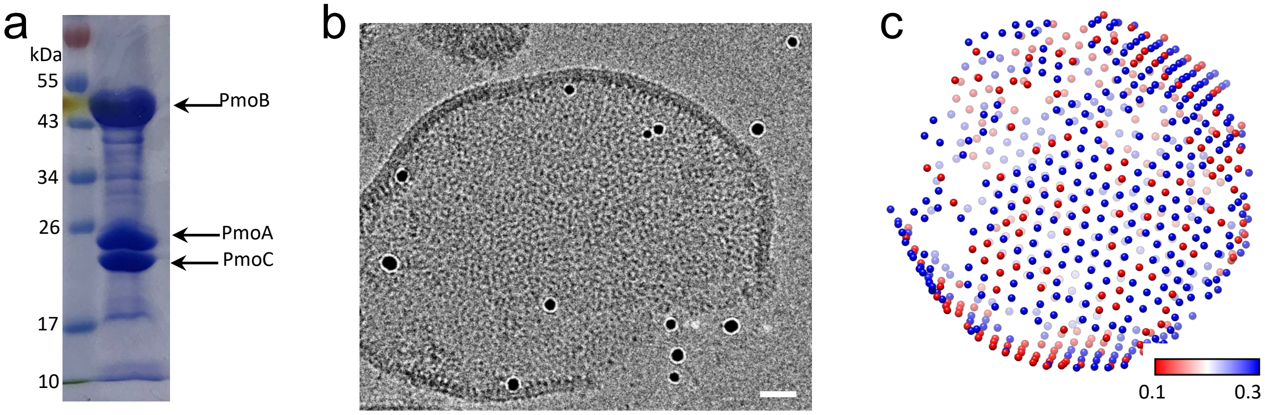

Supplementary Figure 1 | CryoET of isolated ICMs. (a) SDS-PAGE of isolated membranes containing pMMO. The pMMO subunits (labeled) are the most abundant proteins. Similar results were obtained in three independent experiments. (b) A projection of isolated ICM (from n=187). (c) emClarity template matching results using the 7-particle unit as a template, colored by the cross-correlation values (0.1-0.3) from highest (blue) to lowest (red). Scale bar, 50 nm.

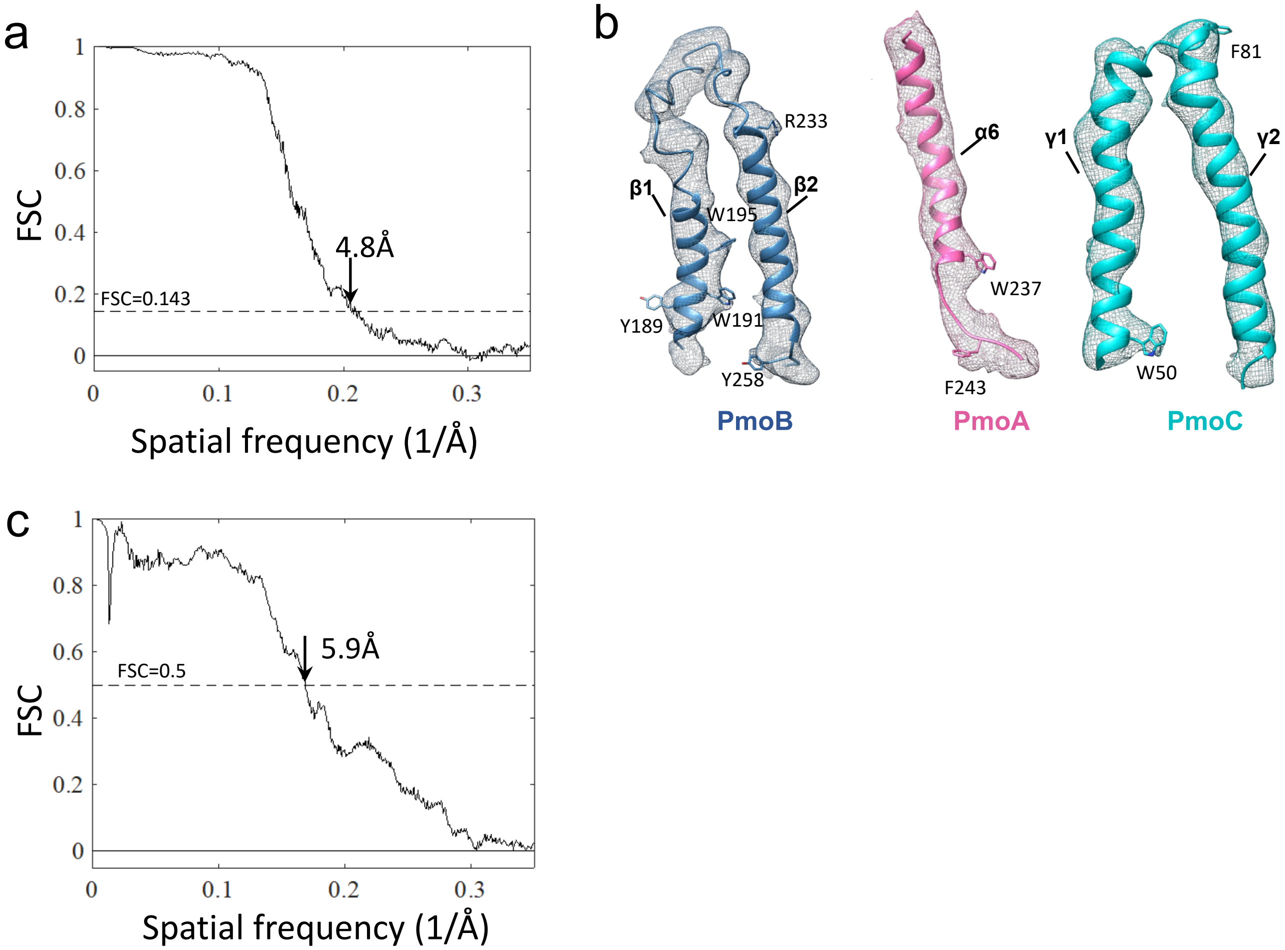

Supplementary Figure 2 | CryoET STA of pMMO trimer. (a) Fourier Shell Correlation (FSC) plot of pMMO trimer. The resolution is indicated at the FSC value of 0.143. (b) Representative helix densities from PmoA, PmoB, and PmoC, superimposed with the crystal structure model (PDB 3RGB), with some bulky amino acids labeled. (c) Fourier Shell Correlation (FSC) plot of pMMO trimer map with pMMO structural model from nanodisc (PDB 7S4H).

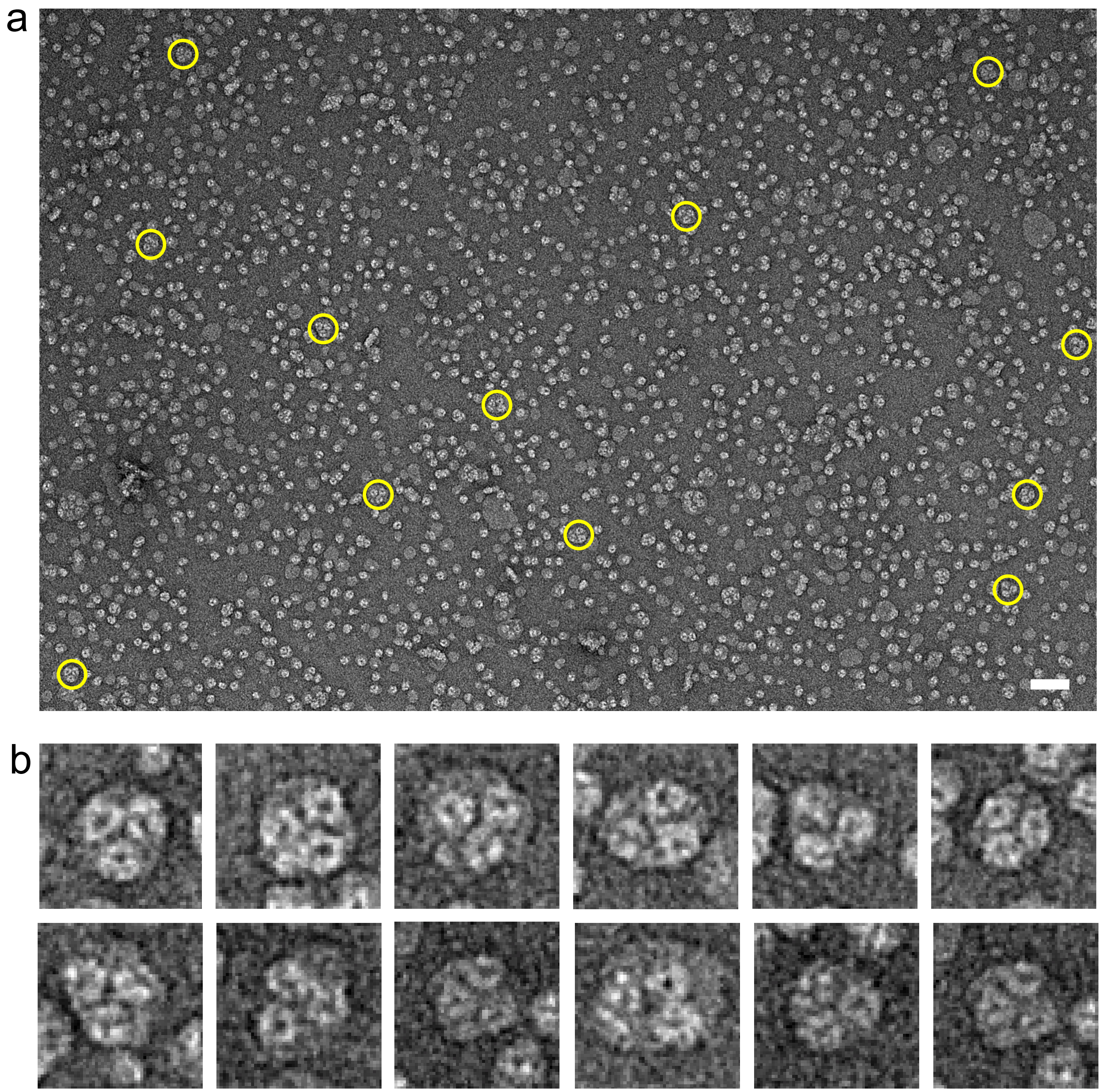

Supplementary Figure 3 | EM characterization of pMMO reconstituted in lipid nanodiscs. (a) A projection image of negatively stained pMMO particles in MSP2N2 nanodiscs. Circled nanodiscs contain three pMMO particles. Similar micrographs were obtained in three independent experiments. (b) A gallery of enlarged views of the circled nanodiscs. Scale bar, 50 nm.

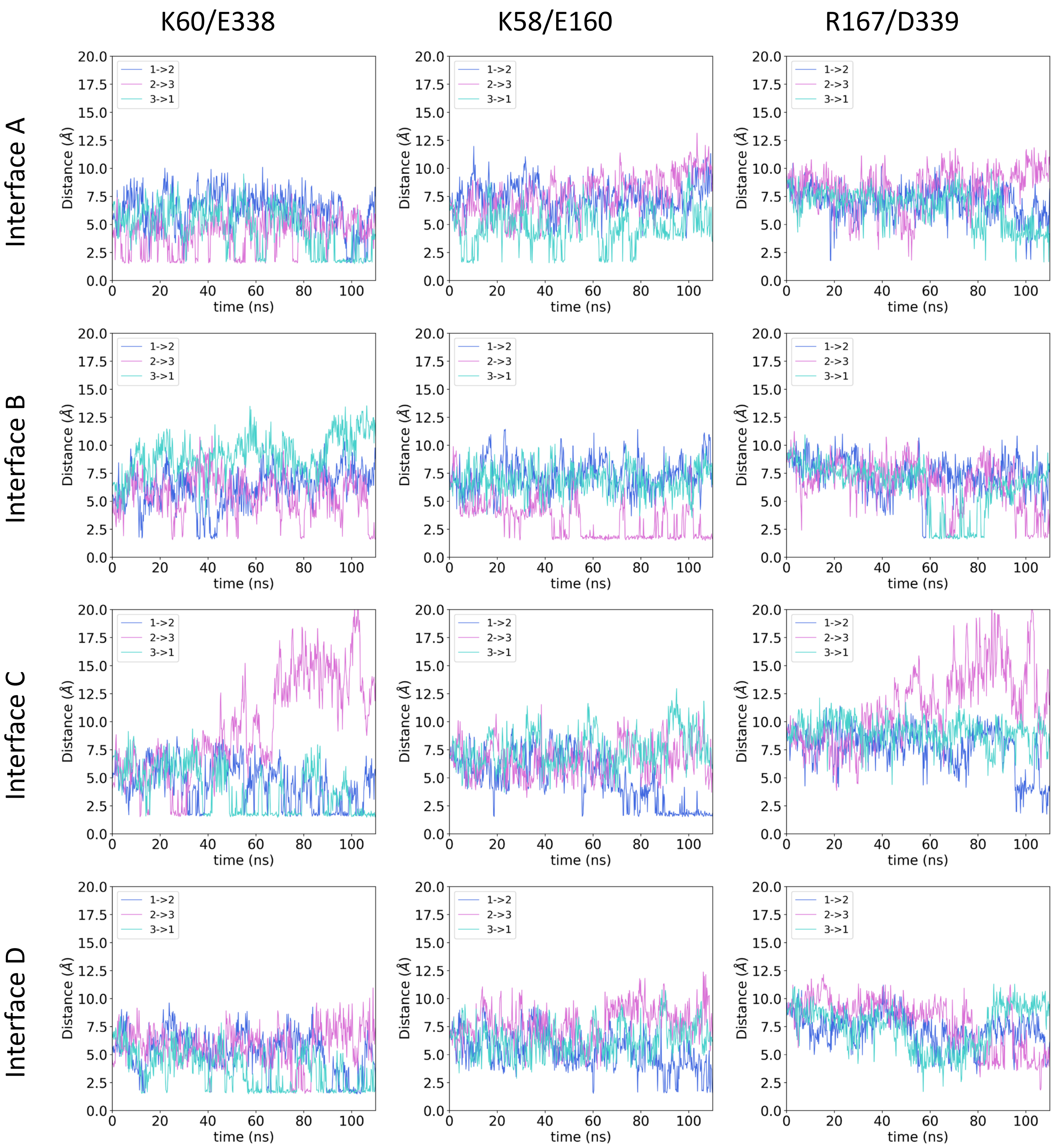

Supplementary Figure 4 | Distance timeseries for highlighted residue pairs at trimer interfaces. Minimum distance between K60/E338, K58/E160 and R167/D339 residue pairs at each of the four trimer interfaces over the course of a 100-ns MD simulation. For each residue pair, the traces for the three pairwise interactions at a given interface are shown as blue, teal, or pink. A prolonged distance of less than roughly 3.5 Å indicates a strong interaction.

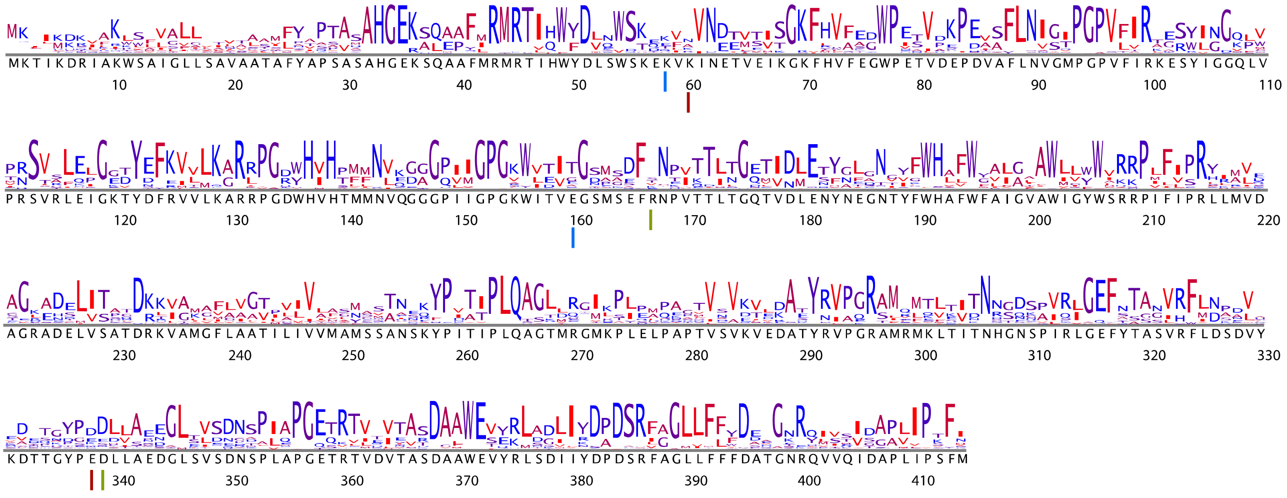

Supplementary Figure 5 | Sequence conservation logo for PmoB from Type I methanotrophs. The logo was generated from 175 sequences from alphaproteobacterial methanotrophs aligned using a hidden Markov model from pfam PF 04744. Residues are colored by hydrophobicity with blue as hydrophilic and red as hydrophobic. Residues that are modeled as participating in intertrimer salt bridges are labeled by matching colored lines (blue, red, green). The sequence shown in black is of PmoB from *M. capsulatus* (Bath).
