# Supplementary material for: Structure and activity of particulate methane monooxygenase arrays in methanotrophs
Source: Nat Commun. 2022 Sep 5;13:5221. doi: 10.1038/s41467-022-32752-9 (PMC9445010; doi:10.1038/s41467-022-32752-9)
Supplement: Supplementary file 2 — Description of Additional Supplementary Files [file 41467_2022_32752_MOESM2_ESM.pdf]

Description of Additional Supplementary Files

File Name: Supplementary Movie 1
Description: The reconstructed 3D volume by Serial cryoFIB/SEM of intact cell cultured without copper

File Name: Supplementary Movie 2
Description: The reconstructed 3D volume by Serial cryoFIB/SEM of intact cell cultured with 25 μM CuSO4.

File Name: Supplementary Movie 3
Description: Reconstructed 3D cryo-tomogram of the cell lamella (with segmentation) shows membrane connectivity.

File Name: Supplementary Movie 4
Description: Reconstructed 3D cryo-tomogram of the cell lamella that were milled along the ICMs.

File Name: Supplementary Movie 5
Description: Reconstructed 3D cryo-tomogram of isolated ICMs.

File Name: Supplementary Movie 6
Description: pMMO cryoET STA map overlaid with the model of pMMO nanodisc structure (PDB 7S4H).
